# Practical Notes

**Published:** 1890-08

**Authors:** 


### PRACTICAL NOTES.

### NEURALGIA.

*By Dr. W. M. Hightower, Sheridan, Ark.*

℞. Tinct. hyoscyamus, ʒ ij.

Bromide ammonia, ʒ j

Salicylate soda, ʒ j.

Water, q. s., ʒ iv. M

Sig. Teaspoonful every half hour until relief is obtained, or four doses are taken.

### GARGLE FOR SUBACUTE PHARYNGITIS.

*By Dr. F. W. Mittendorff.*

℞. Ferri et ammonii aulph. (U. S. P),
Potassii chloratis, aa dr. 1
Aquæ, fl. oz. 16. Dissolve.

Sig. Use as a gargle, morning and evening.

### HELIOTROPE PERFUME.

Mr. E. Campe proposes the following formula in the *Chemiker and Druggist* for a perfume for retail sale:

Oil of bergamot, 1 1-2 oz.
Vanilla, 8 grs.
Tincture of benzoin, 2 drms.
Rectified spirit, 60 oz. Solve.

### WARTS.

*By Dr. J. M. White, Pleasant Ridge, Ala.*

℞. Ung. pyrogallic, 20 per cent., ℥j.

Sig. Apply 2 or 3 times a day.

---

TO STOP THE SECRETION OF MILK.—Dissolve one half ounce camphor in twelve ounces of turpentine, and apply to the breasts when desiring to stop the secretion of milk.—*Medical Review.*

The following mixtures will prove of benefit in the treatment of intractable cases:

LOOMIS' DIARRHŒA MIXTURE.

| | |
|---|---|
| Tincture of opium, | 1-2 fl. oz. |
| Tincture of rhubarb, | 1-2 fl. oz. |
| Compound tinct. catechu (U. S. P.), | 1 fl. oz. |
| Oil of sassafras, | 20 minims. |
| Compound tincture of lavender, enough to make | 4 fl. oz. |

M. Sig. One teaspoonful every four hours, for adults.

SQUIBBS' DIARRHŒA MIXTURE.

| | | |
|---|---|---|
| Tincture opium, | 1 | fl. oz. |
| Tincture capsicum, | 1 | fl. oz. |
| Spirit of camphor, | 1 | fl. oz. |
| Purified chloroform, | 180 | minims. |
| Alcohol, enough to make | 5 | fl. ozs. |

M. Sig. One teaspoonful every five hours, for adults.

RINGWORMS.

*By Prof. Shoemaker, Philadelphia.*

Two cases of ringworm on the head and face were cured by an ointment:

℞. Cupri oleati, ʒ ss.

Adipis benzoati, ℥ j.

Mice, flat unguent, et signe: Use locally.

DYSMENORRHŒA.

℞. Bromide of ammonium, ʒ ij.

Bromide of potassium, ʒ iv.

Aromatic spirit of ammonia, fʒ vj.

Camphor-water sufficient to make fʒ vj. M.

Sig. Of this, from a dessertspoonful to a tablespoonful may be given every two or four hours.

FOR BURNS AND SCALDS.

*By Prof. Bartholow.*

℞. Acidi salicylici, dr. j.
Olei olivæ, fl. oz. viij. M.

Sig. Apply to burn, covering with linen or lint.

COUGH MIXTURE.

*By Dr. E. G. Janeway.*

℞. Syr. tolutani,
Syr. pruni Virginianæ,
Tinct. hyoscyami,
Spir. ætheris comp.,
Aquæ, aa part æquales. Mix.

Dose. A teaspoonful.

INK AND RUST STAINS

Are removed easily by a solution containing ten parts each of tartaric acid, alum, and distilled water. The solution has the trade name of "encrivoir."—*Illus. Med. Jour.*

---

THIELMANN'S DIARRHŒA MIXTURE.

| | |
|---|---|
| Wine of opium, | 1 fl. oz. |
| Tincture of valerian, | 1 1-2 fl. oz. |
| Ether, | 1-2 fl. oz. |
| Oil of peppermint, | 60 mins. |
| Fluid extract of ipecac, | 15 mins. |
| Alcohol, enough to make | 4 fl. ozs. |

M. Sig. Thirty drops every three to five hours for adults. —*College and Clinical Record.*

---

In gonorrhœa Julien (*Revue de Therap.*, March 15th, in *Med News*, April 5th) recommends the following injection:

| | | |
|---|---|---|
| R | Liquid vaseline, | p. 140. |
| | Bismuth subnitrate, | p. 10. |
| | Resorcin, | p. 3. |
| | Iodol, | p. 1. |
| M. | | |

---

THE CODE OF ETHICS IN OREGON.—In a bill before the Oregon Legislature, for the regulation of medical practice there is a singular clause providing that licenses to practice may be revoked for "unprofessional conduct," which is defined in the bill as: (1) Participation in criminal abortion; (2) employing "drummers or steerers;" (3) promising to cure an incurable disease, and taking a fee therefor; (4) betrayal of a professional confidence; (5) advertising in a false or exaggerated manner;

(6) advertising to regulate the menses of women; (7) conviction under some charge implying "moral turpitude;" (8) habitual intemperance. Like many other newly settled communities, Oregon has greatly suffered from quacks and advertising adventurers.—*Medical News.*

---

The health officer of Chicago has refused to accept "heart-failure" as a cause of death. It is said that one hundred and fifty death certificates so signed have been returned, with a request for information as to the true cause of death.—*Times and Register.*

We do hope our board of health will do likewise.

---

According to Dr. L. G. Doane, of New York, a neat way of giving quinine is as follows:

| | | |
|---|---|---|
| ℞ | Quinine, | 1 grain. |
| | Chocolate, | 1 scruple. |

Children, he says, will take quinine in this form, and cry for more.—*Buffalo Med. and Surg. Journal.*

---

TREATMENT OF ERYSIPELAS.—Dr. Koch treated numerous cases of erysipelas with the following ointment:

| | |
|---|---|
| Creolin, | ʒi. |
| Iodoform, | ʒiii. |
| Lanolin, | ℥i. |

This ointment is spread as an even, smooth layer over the affected skin and its surroundings, on an area of at least two to three inches to the outside of the inflamed parts. The whole is covered by a piece of mackintosh. Dr. Koch selected creolin in the above prescription because he thought that it was possessed of first-class disinfectant properties, without sharing the dangerous after-effects of carbolic acid. Iodine, which is derived from the decomposition of iodoform, stimulates absorption of inflammatory products. Lanolin has been chosen because it penetrates the skin best of all ointment bases.—*American Practitioner and News.*

---

Solution of chloral hydrate, grains five to the ounce of water, will clear the hair of dandruff and prevent it falling out from this cause.—*American Lancet.*

ANTIMONY IN INFLAMMATIONS.—In the *Practitioner* for March, 1885, Dr. Spender pointed out that antimony, in frequently repeated small doses, one-sixth of a grain of tartar emetic, every hour or two hours, has the power of completely dissipating early local inflammations. Acting on this suggestion, Surgeon-Major Lawrie began the use of small and frequently repeated doses of antimony in the treatment of surgical inflammation at the Afzalgunj Hospital in May, 1885. Since then its use has been gradually extended, and he has come to look upon it as one of the most valuable of drugs, as useful in local inflammations as quinine is in malarious fever. It is now used at the Afzalgunj Hospital in all inflammatory diseases which are not of a specific nature.

It may be given without fear of causing nausea and diarrhœa or depression, even in diseases where its use would appear to be contra-indicated—for example, in mucous enteritis, a most fatal disease to children on the plains of India. It has been employed in the treatment of typhoid fever, and is said to cut the disease short in a most remarkable way.

Tolerance of the drug is very soon established; there is no depressing effect unless it is pushed so far as to cause its own peculiar nausea and diarrhœa. It can be administered with cardiac tonics, and there are few, if any, cases which are susceptible of benefit by it in which it cannot be employed in sufficient quantity to do good without fear of inducing depression. —*American Journal of Medical Science.*

---

LAW AGAINST ADULTERATION IN RUSSIA.—The Russian Government has recently enacted some very stringent laws against the adulteration of food and drink. Any person guilty of adulterating any article of food will be liable to a fine of $200 or imprisonment for three months for the first offence, double this penalty for the second, and deprivation of all rights as a citizen for the third.

---

A JUST OBSERVATION.—The man who remains abstemious where no liquor is to be had does not deserve much credit, but the man who is temperate when the sparkling champange stands beside his plate merits our approbation.—*Journal of the American Medical Association.*

WHOOPING-COUGH TREATED WITH TERPINE HYDRATE.—Manasse (*Therap. Monatshefte*, 1890, 116) gives some account of the constitution of terpine hydrate and of the uses for which it has been recommended, and then reports his own experience with it in the treatment of whooping-cough, having tried it in 41 cases with excellent results. In children less than one year old it may be given in dose of 22 grains per day without any deleterious effects. A careful history of each case was kept, the number of paroxysms being noted daily. In the great majority of cases 22 to 45 grains daily were sufficient in the course of four to five days greatly to reduce the number, or, at any rate, the severity of the attacks; and to hasten recovery from the attending bronchitis.

In endeavoring to explain the method of its action, he discards at once the former view, that pertussis is in any sense a neurosis of the pneumogastric or phrenic, accepting the theory that the disease is of mycotic origin. As terpine hydrate has been shown by Colpi to possess quite powerful antiseptic properties, we might assume that the value of the drug depended upon its action upon the germs of pertussis. The author is, however, of the opinion that little is ever to be gained by the attempt to cure any infectious disease by attacking the germs themselves, since these bodies are much less apt to be injured by drugs than are the tissues of the human organism. In pertussis we have an inflammatory and catarrhal condition of the respiratory mucous membrane, which, in the severe cases, may increase to a grave condition, blocking the capillary bronchi with mucus and cellular masses, and producing alveolar collapse, lobular hyperæmia, degeneration of the terminal bronchioles and death. Terpine hydrate does good by causing a contraction in the blood-vessels, and thus producing an anæmia of the respiratory mucous membrane with diminution of its swelling and increase of the secretion.—*American Journal Medical Science.*

---

A BALTIMORE boy is said to have suffered a unique accident. While drinking coffee from a flask his tongue was drawn into the flask by suction, and, becoming fast, swelled, and required the services of a physician to release it. That boy certainly had a pretty strong breath.—*Times and Register.*

THE TREATMENT OF CLUB-FOOT.—Orthopedic surgeons are by no means in accord as to the treatment of talipes, some advocating tenotomy in every case, others regarding operative measures as the last resort, and to be thought of only after a long trial of stretching. In the April number of the *American Journal of the Medical Sciences*, Dr. James K. Young reviews the testimony on either side, and comes to the conclusion that there is no arbitrary rule to be observed. He sums up the indications for one or the other method as follows: 1. Stretching should always be first employed in cases which are seen early. 2. Mild cases should always be treated by stretching. 3. Patients who object to the knife can be affectually cured by stretching, but it requires a longer time. 4. A tenotomized tendon is as strong as one that has been stretched. 5. In severe cases it is better to save time by tenotomy. 6. After treatment to prevent relapse is quite as important as the operation itself.—*Medical Record.*

---

THE MAIN OBJECT.—Is the listening to papers and discussions the end of chief importance in attending a session of the American Medical Association? We think not. Are we not better able to read and comprehend these, to digest and absorb at our leisure what we like of their scientific lore as they appear in the Association Journal, than when they are sprung on us in this hasty fashion, when we may be impressed more by the manner of the speaker than by the soundness of his argument?

We believe that one of the greatest benefits to be derived from such meetings is the acquaintance which we form with those whose names and writings are already familiar to us. There we meet the *men*, and not the writers. We study their learning, their appearance, their very selves, in fact, and the groundwork for a better appreciation of their true worth is thus given us. An acquaintance with the members of this body is no small possession; it is a tangible reality, of which the possessor may well be proud. There are congregated the venerable fathers of medicine and surgery of our country—those who have labored long and earnestly in the great cause of the advancement of the profession, and for the concentration and organization of its forces; and who yet stand in the traces lending their aid with fatherly beneficence. There we see the *workers* of the profession, the men who originate, who record, who develope and formulate the rules and principles of medical and surgical practice, who, in their turn, at some future date, will be looked upon as the fathers of the profession—but who, no doubt, will resent a too early application of that term, or its application in too comprehensive a sense.—*Weekly Medical Review.*

---

INFLUENCE OF COUGHING IN THE REDUCTION OF HERNIAS.—Dr. Vandenabeele, *Gaz. m. de Strasbourg*, *Lond. M. Rec.*, January. The author was called to see a man with left inguinal hernia. Had pulmonary emphysema for years; the severe coughing had caused the hernia, which at last became strangulated. It was of the size of a small orange, and after applying taxis for twenty minutes no result was obtained. Suddenly, in spite of interdiction, the man coughed violently, but the hernia was not released from pressure; a gurgling was heard meanwhile, and the sac was half emptied. After a minute's rest the hernia was again seized, and the man was directed to cough. It was reduced at once. He began to suspect that the act of coughing caused dilatation of the inguinal ring, and awaited another case. Case 2. Some months afterwards he was called to a woman with left femoral hernia, and found Dr. Cohen there. The hernia was the size of a large nut, and taxis had been tried half an hour. His *confrere* advised sending her to hospital, but he requested a trial. Two minutes taxis gave no result, after which he said: "Now for my method," and holding the hernia firmly, asked the patient to cough strongly. A gurgling noise was heard at once, and the hernia was reduced, to the astonishment of his *confrere*. Case 3. A short time afterwards he was called to a concierge, who had just been visited by a *confrere*, on account of strangulated inguinal hernia. After taxis by the latter for half an hour transport to the hospital had been advised. After taxis for a short time, which gave no result, he adopted the above method, and the hernia was instantly reduced. During five years he has in this way reduced four crural hernias in women, and seven inguinal hernias in men, besides the three cases mentioned. One patient only has been sent to hospital—an old man of eighty years who could not cough. He concludes that both the inguinal and the crural rings are temporarily dilated by the act of coughing, and if pressure be made at the same time, the compressed gases escape into the abdominal intestine. He believes he has discovered an easy method of reducing hernias, superior to any method hitherto suggested.—*N. Y. Med. Abstract.*

